# Characteristics and temporality of the ventilatory techniques in the management of acute respiratory distress syndrome: A scoping review

**DOI:** 10.2478/jccm-2025-0019

**Published:** 2025-04-30

**Authors:** Théo Battalian, Raúl Escudero Romero, Arianne Barzaga Molina

**Affiliations:** Department of Physiotherapy, School of Medicine, Universidad San Pablo-CEU, CEU Universities, Boadilla del Monte, Spain; Department of Kinesiology, Faculty of Medicine, Clínica Alemana Universidad del Desarrollo, Santiago, Chile

**Keywords:** acute respiratory distress syndrome (ARDS), oxygen therapy, high-flow nasal cannula (HFNC), non-invasive ventilation (NIV), invasive mechanical ventilation (IMV), mechanical ventilation, temporality, critical care, intensive care unit (ICU), patient-centred care

## Abstract

**Introduction:**

Acute Respiratory Distress Syndrome (ARDS) is a critical condition characterised by acute respiratory failure due to increased alveolar-capillary membrane permeability. This leads to non-cardiogenic pulmonary oedema, hypoxemia, and impaired respiratory compliance, significantly impacting patients’ survival and quality of life. The management of ARDS involves various ventilatory and non-ventilatory therapies. Understanding the optimal timing and application of these therapies is crucial for improving patient outcomes.

**Aim of the study:**

This scoping review aims to identify and synthesise the ventilatory techniques used in managing ARDS, focusing on their temporality and the interplay between different therapies. The study seeks to synthesize the available evidence and summarize current management strategies, highlighting areas for further research and improvement in ARDS care.

**Material and Methods:**

A systematic search of PubMed, EBSCO, and ScienceDirect databases was conducted, following the Joanna Briggs Institute guidelines (2015), for articles published between 2013 and 2023. Studies involving adult patients (18 years or older) diagnosed with ARDS and receiving ventilatory support in the ICU were included. Exclusion criteria included other acute respiratory pathologies, clinically extreme obese patients, and patients with tracheostomy.

**Results:**

437 articles were identified through the database search, of which 23 met the inclusion criteria and were included in the final review. Most articles were published between 2015–2019 (43.5%), originated from the USA (34.78%), and employed observational study designs (73.91%). The included studies reported on patients aged between 23 and 79 years, with intrapulmonary causes being the most common aetiology for ARDS. Various ventilatory strategies were identified, including conventional oxygen therapy, high-flow nasal cannula (HFNC), non-invasive ventilation (NIV), invasive ventilation (IMV), and combined approaches. Temporality was reported in 35% of the articles, but none of them as their primary focus.

**Conclusions:**

The review highlights the diversity of ventilatory techniques employed in ARDS management and the importance of individualizing treatment strategies based on patient response and disease severity. The temporality of these interventions remains a crucial aspect, requiring further investigation to establish clearer guidelines for optimizing the timing and sequence of ventilatory support in ARDS. The findings underscore the need for future research to focus on patient-centred outcomes and the long-term implications of ARDS management, including quality of life and functional status.

## Introduction

ARDS is a syndrome characterised by acute respiratory failure due to increased permeability of the alveolar-capillary membrane, leading to non-cardiogenic pulmonary oedema, hypoxemia, and respiratory system compliance impairment [1,2,3]. The pathophysiology of ARDS is complex and involves various inflammatory mediators and cellular mechanisms. This inflammatory process is characterised by rapid and severe lung inflammation that damages the alveolar epithelial cells and pulmonary microvascular endothelial cells, resulting in diffuse alveolar damage (DAD). This damage causes an initial exudative phase characterised by high-permeability, proteinaceous pulmonary interstitial, and alveolar oedema, along with the injury and death of endothelial and alveolar epithelial cells. This leads to a delayed fibroproliferative phase comprising fibrosis in intraluminal and interstitial compartments and type II alveolar epithelial cell proliferation [4].

ARDS is classified according to the Berlin classification introduced in 2012 [5]. It is composed of 4 parameters: timing, radiographic criteria, origin of oedema and oxygenation. The Berlin criteria provide more specific guidelines for defining ARDS severity based on the PaO_2_/FiO_2_ ratio. Additionally, a minimum positive end-expiratory pressure (PEEP) level of 5 cmH_2_O is required for diagnosis.

Treatment options are wide since they are implying the concomitant use of both ventilatory and non-ventilatory techniques. There are multiple options under the umbrella of ventilatory therapies such as high flow nasal canula (HFNC), non-invasive ventilation (NIV) and invasive ventilation (IMV). On the other side, among the non-ventilatory therapies it is important to cite the use of prone positioning (PP), recruitment manoeuvres and the use of neuromuscular blockade.

Regarding the use of ventilatory therapies, HFNC can be used first-hand. The high flow rates and humidification makes it a comfortable and well-tolerated alternative to conventional oxygen therapy or NIV. The use of HFNC is based on the principle that high flow rates and humidification can improve gas exchange, reduce work of breathing, and to prevent atelectasis by using PEEP [6,7].

On the other hand, NIV is a respiratory support technique that aims to deliver positive pressure to the lungs without the need for an endotracheal tube. It is typically delivered through a mask, nasal prongs or a helmet and the positive pressure helps to improve oxygenation and reduce carbon dioxide retention. The increased lung volume and oxygenation can help to reduce the work of breathing and alleviate respiratory distress [1,8]. NIV can have several positive effects in patients with respiratory failure [6,9,10,11]; it improves oxygenation by reducing shunt fraction and increasing functional residual capacity; reduces the work of breathing by offloading the respiratory muscles; improves gas exchange by enhancing alveolar ventilation and CO_2_ clearance; prevents the need for ETI and mechanical ventilation; improves patient comfort and reduces anxiety. There is already strong evidence for NIV in case of exacerbation of Chronic Obstructive Pulmonary Disease (COPD) [12] and for cardiogenic oedema [13]. However, about ARDS, the level of evidence is still to be proven.

The most invasive ventilatory option for the treatment of ARDS is endotracheal intubation and subsequent use of IMV. It has been a topic of extensive research [6,14] and debate in recent years, with the goal of optimising patient outcomes and minimising complications. IMV aims to improve oxygenation, decrease carbon dioxide levels, and decrease the work of breathing, thereby reducing respiratory muscle fatigue. However, it can also lead to complications, such as barotrauma, ventilator-associated pneumonia, and hemodynamic instability. Therefore, it is crucial to balance the potential benefits of IMV with the risks of complications when deciding whether to initiate it [6,9,15,16,17,18]. Additionally, ETI results are based on when it is performed. If delayed, mortality has been shown to increase significantly [19,20]. If premature, both short- and long-term side effects for patients will be amplified [10,21]. Various strategies have been developed to optimise the use of IMV in ARDS patients. They are named the protective ventilation strategies: Low tidal volume: 4 – 8 ml/kg of predicted body weight or less; inspiratory plateau pressure < 30 cm H_2_O; applying appropriate PEEP; driving pressure < 15 cm H_2_O; respiratory rate control to avoid fatigue and complication; mild permissive hypercapnia in order to avoid high tidal volumes in some cases; periodic recruitment manoeuvres to open collapsed lung regions and improve oxygenation [8,22].

Additionally, the combination between ventilatory and non-ventilatory techniques is crucial to provide the patient with optimal care in the management of ARDS. In the scope of non-invasive therapies, PP relies on solid evidence. It is a technique that has been used for many years to improve oxygenation in patients and has been the subject of far-reaching research. Solid conclusions have been drawn upon the results of the latter. Two randomized control trials (RCT) are the bases of the advancement of this technique in improving both oxygenation and decreasing mortality [23,24]. PP allows a homogenisation of the pressure gradient in relation to the redistribution of the infiltrated areas and the abdomen cranial shift; decreases the weight of the cardiac mass and permit the lung elastance to increase, thus letting the respiratory system elastance return towards base-line values. Mortality is the primary outcome that is modified when applying PP. Guerin et al. are showing a 16,8% decrease between the prone and supine group in their RCT [23].

Recruitment therapy manoeuvre is a pivotal intervention in the management of ARDS, aimed at optimising lung function and improving oxygenation. It involves the application of controlled PEEP to the respiratory system, with the objective of opening collapsed or poorly aerated alveoli within the lungs. Several strategies for recruitment therapy are utilised, including the staircase, sustained inflation, and decremental methods. The choice of technique depends on individual patient factors, such as lung compliance, severity of ARDS, and the presence of comorbidities. One of the primary advantages of the therapy is the improvement in oxygenation and lung compliance. The RCT conducted by Meade et al. demonstrated improved oxygenation and reduced mortality rates in patients subjected to recruitment manoeuvres [25,26,27,28].

Lastly, neuromuscular blockade is a technique used in the case of patient-ventilator asynchrony. It aims to reduce the risks of patient-induced lung injury (PILI) or ventilator-induced lung injury (VILI) and lead to better patient tolerance of mechanical ventilation, which often requires the use of high levels of positive pressure to maintain oxygenation and ventilation. However, the use of neuromuscular blockade in ARDS is a complex and controversial topic, and its potential benefits must be weighed against the risks. Complications such as ICU acquired weakness, protective reflexes inhibition and ethical dilemmas are at the core of the debate on whether it should be applied [29,30].

While the immediate goal of ventilatory support is to improve oxygenation and reduce the work of breathing, it is essential to consider the potential long-term effects on patients’ overall health and quality of life. This includes the risk of developing Post-intensive care syndrome (PICS). Post-intensive care syndrome (PICS), which is defined as a group of problems that people can experience after surviving a life-threatening illness [31] has been studied and affects post-ICU patients with changes in lung function, reduction in quality of life and functional status, and the appearance of neuropsychiatric disorders up to 5 years after their critical illness [32]. Likewise, quality of life is a concept that needs to be considered. and take into account when deciding the treatment plan. Davidson et al. and Martí et al. have shown that 1-year post-ICU survivors had a significant decrease in this area, specifically marked among younger patients [32,33].

The selection and timing of ventilatory support can have a profound impact on patients’ long-term quality of life. Early use of less invasive techniques, such as HFNC or NIV, may help to avoid the complications associated with invasive mechanical ventilation and improve long-term outcomes. European and Japanese medical associations [6,34] are concomitant on the use of PaO_2_/FiO_2_ ratio as the number one parameter to determine the treatment line. However, when analysing guidelines, it is noticeable that the concept and use of temporality lacks. Knowing which therapy to use is key. It is as significant to determine when to do so.

Therefore, the following exploratory review is proposed, whose main objective is to describe the ventilatory techniques used in the management of ARDS and its temporality. The foregoing, in order to synthesise the available evidence in this regard and summarise the different management strategies for this pathology. Additionally, this review aims to regroup variables such as ventilatory administration time, ventilation parameters, mortality rates and quality of life on a broad timing perspective.

## Material and methods

### Study design

An exploratory review of the literature (Scoping Review) was carried out in different scientific databases (PubMed, EBSCO and Science Direct) using relevant search strategies for each of them; in order to describe the ventilatory techniques used in the management of ARDS and its temporality (see supplementary material). Using the framework proposed by the 2015 Johanna Briggs Institute Manual. As this study involved a review of published literature and did not involve human subjects, ethical approval was not required.

### Research question

What are the ventilatory techniques used in the management of ARDS, its characteristics and temporality?

The inclusion criteria for this review encompass adults aged 18 years or older diagnosed with ARDS, specifically within the ICU population. The focus is on the management of ARDS, considering any type of articles published between 2013 and 2023.

Articles were excluded if they involved patients with other forms of acute respiratory failure, clinically extremely obese individuals (BMI > 35 kg/m^2^), or pregnant populations. Additionally, studies that did not address ventilatory management of ARDS, involved patients with tracheostomies, or lacked available full-text versions were not considered.

### Study selection

The articles obtained from the search strategy in each of the databases were transferred to a matrix in Rayyan^®^, where duplicate articles were first identified and eliminated. Subsequently, the article selection process included the application of the inclusion and exclusion criteria in three review stages: by title, abstract, and full text, performed by two investigators. A third researcher defined the inclusion of articles in case of disagreement between the first two.

### Data extraction

The selected articles were exported to a Microsoft Excel^®^ spreadsheet for the extraction of information regarding bibliometric variables such as title, author, year of publication, language, study design, country of publication, and variables of interest referring to ventilatory management, timing of application of therapies and reported outcomes. A quality control was carried out at this stage, where each researcher again extracted information from 22% of the articles that the other researchers had already reviewed, thus ensuring the correct management of the information and avoiding its loss.

## Results

### Article selection process

The updated preferred reporting items for systematic reviews and meta-analyses (PRISMA) has been used to report the following study.

**Fig. 1.**
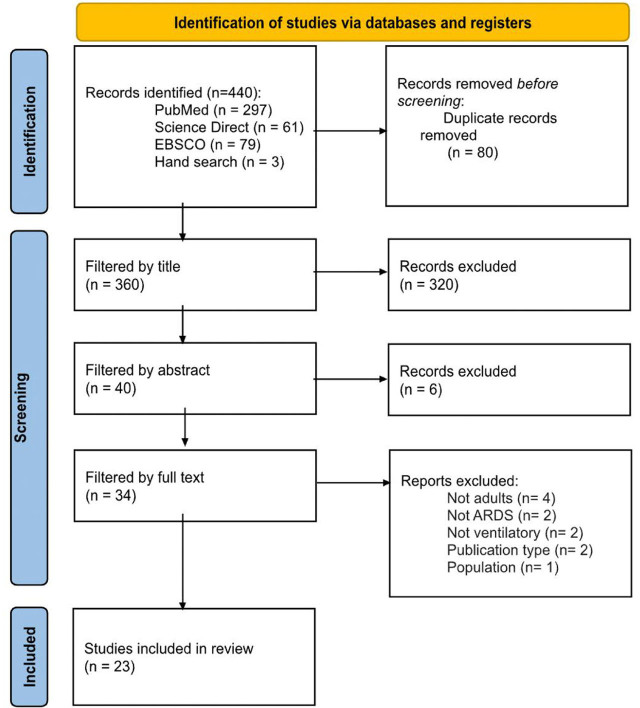
**Flowchart of the study selection process following the PRISMA guidelines.** (ARDS: Acute Respiratory Distress Syndrome; EBSCO: Elton B. Stephens Company)

After performing the bibliographic search in the databases (*Supplementary material*) a total of 437 articles were retrieved and three other articles were included by hand search. The search for duplicates led to the removal of 80 articles. The first-pass screening was conducted through titles to identify those manuscripts that were not relevant to the question, which removed 320 articles. The abstracts of 40 articles were screened, leading to the removal of six irrelevant articles. Lastly, the full text of 34 articles were then assessed for eligibility, from which 11 got removed. Therefore, a total of 23 studies were included in the review. The complete PRISMA flow chart is represented in *Figure 1*.

### Population characteristics

The total number of patients with ARDS diagnosis included in all articles selected in this review was 19,113, with an age range between 23 and 79 years. Etiological factors contributing to ARDS varied within the cohort, with most of the patients referring to intrapulmonary causes.

### Bibliometric variables

The articles were published over three periods, with the majority (43.5%, n=10) published between 2015–2019, followed by 39.13% (n=9) in 2020–2023, and 17.39% (n=4) in 2010–2014. Regarding the country of origin, the United States had the highest representation of publications, accounting for 34.78% (n=8) of the total, followed by the United Kingdom (26.09%, n=6), Switzerland (8.7%, n=2), and a collective representation of 30.43% (n=7) from diverse other nations. As for the study designs, seventeen studies were observational (73.91%, n=17), five articles were experimental (21.74%, n=5) and only one review article (4.35%, n=1) (Table 1).

**Table 1.** Bibliometric variables

	**Number of articles (%)**
**Year of publication**	
2010–2014	4(17,4)
2015–2019	10(43,5)
2020–2023	9(39,1)

**Country of publication**	
United States	8(34,8)
United Kingdom	6(26,1)
Switzerland	2(8,7)
Others	7(30,4)

**Study design**	
Review	1(4,4)
Observational	17(73,9)
Experimental	5(21,7)

**Ventilatory strategies**	
Conventional oxygen therapy	3(13,1)
High Flow Nasal Cannula	2(8,7)
Non-invasive ventilation	7(30,4)
Invasive ventilation	17(73,9)
Combination of strategies	4(17,3)

### Ventilatory therapies and temporality

Regarding ventilatory management, there are various reports about type of therapy, complexity, and temporality; sometimes they could even coexist. The therapies reported in the articles included in this review correspond to oxygen therapy, high-flow nasal oxygen therapy, NIMV, and IMV, in addition to the use of high-frequency oscillatory ventilation (HFOV) being reported in one article. The most frequently reported ventilatory therapy in the treatment of ARDS was IMV with 74% (n=17), and the least frequent was HFNC with 8.7% (n=2), and the most frequently recorded ventilatory parameter was PEEP with 70% (n=16), followed by oxygen concentration with 57% (n=13). In relation to the use of non-ventilatory therapies, the use of neuromuscular blockade and prone position were reported with the same frequency (see Table 2). Regarding the temporality of the ventilatory therapies applied, the articles that mention the coexistence or use of more than one ventilatory therapy for the management of ARDS, for the most part, do not detail the time of use of each therapy separately; only one article (4.3%) details it independently.

**Table 2.** Report of ventilatory record

	**Number of articles (%)**
Coexistence of ventilatory strategies	5 (22)
PEEP evaluation	16 (70)
Evaluation/record of pulmonary mechanic parameters	11 (48)
Report of the time of use of each strategy	21 (91)
Report of intervention with Oxygen therapy	13 (57)
Report of prone position as a non-ventilatory strategy	9 (39)

PEEP= positive end-expiratory pressure

**Table 3.** Reported outcomes from the selected articles

	**Number of articles (%)**
**Principal outcomes**	
Reported ARDS severity level (PaO_2_/FiO_2_)	(30,4)
	14(61)
ICU length of stay	11(48)
ICU mortality	10(43)
Duration of ventilation	8(35)
Hospital length of stay	8(35)
Follow-up mortality rate	6(26)
Complications	3(13)
Non-invasive ventilation failure	6(26)
Hospital mortality	14(61)
Other	

**Secondary outcomes**	
Temporality	8(35)
Quality of life	0(0)

**Principal physiological outcomes**	
PaO_2_/FiO_2_	19(83)
pH	4(17)
SaO_2_	3(13)
PaO_2_	2(9)
PaCO_2_	3(13)
Bicarbonate	2(9)
Lactate	2(9)
Oxygen index	2(9)
Other	5(22)

ARDS: Acute Respiratory Distress Syndrome; PaO_2_/FiO_2_: ratio of partial pressure of arterial oxygen to the fraction of inspired oxygen; ICU: Intensive Care Unit; SaO_2_: peripheral capillary oxygen saturation; PaO_2_: partial pressure of oxygen; PaCO_2_: partial pressure of carbon dioxide

### Reported outcomes

According to the outcomes stated in the 23 articles, we could identify 7 articles (30%) reporting ARDS severity level according to the PaO_2_/FiO_2_ ratio. The remaining articles use the PaO_2_/FiO_2_ ratio as a comparison parameter but not to classify the severity of the pathology. The length of stay is the principal outcome that is the most cited in 61% (n=14) of the articles. The second most common outcome reported is the mortality rate as in 48% (n=11) of the papers. Then, 43% (n=10) described the total duration of ventilation.

Temporality was cited as an outcome in 35% (n=8) of the articles. Although, it is important to note that none of the articles had temporality as their primary focus of investigation. Furthermore, none of the articles in the full cohort reported quality-of-life assessments.

Regarding physiological outcomes, PaO_2_/FiO_2_ levels were the most reported, appearing in 83% (n=19) of the articles, followed by pH levels in 17% (n=4), SaO_2_ in 13% (n=3), PaO_2_ in 9% (n=2), and PaCO_2_, bicarbonate, lactate, and oxygen index each in 9% (n=2).

## Discussion

When analysing the studies included in this scoping review, it has been observed that, although the timing of ventilatory strategies is documented in all the studies reviewed, this is not the main study variable. In many cases, intervention times are determined by methodological issues inherent to the clinical trial protocol, rather than by specific adaptation to the clinical needs of the patients. This methodology may limit the direct applicability of the results to real clinical situations, where the timing should be more aligned with the individual evolution of each patient.

In addition, a remarkable heterogeneity has been found in the times reported for the different ventilatory strategies among the studies analysed. This variability may be attributed to both differences in the severity of the patients included in the studies, as well as the diversity in the causes of ARDS reported. For example, while some studies use a PaFi ratio (PaO_2_/FiO_2_ ratio) between 100 and 200 to classify patients as moderate, others use ranges of 150 to 250 for the same purpose. Furthermore, some studies only provided data on whether the patient was classified as ARDS or not ARDS, without considering the severity of the condition. The Berlin classification of 2012 introduced specific severity classifications, which include mild, moderate, and severe categories based on the PaO_2_/FiO_2_ ratio [5]. Patients with a PaO_2_/FiO_2_ ratio of less than 100 mmHg are classified as having severe ARDS. This revision in the assessment tool aims to help clinicians tailor appropriate treatment strategies according to the severity of the syndrome. The inclusion of severity classifications helps in stratifying patients more accurately and potentially improving treatment outcomes. However, this disparity in ARDS severity classifications across studies adds another layer of complexity to comparing results and generalising conclusions. The inconsistency in definitions and severity assessments can lead to significant variations in reported outcomes, making it challenging to draw definitive conclusions from the existing body of research. Therefore, adopting a uniform classification system, such as the Berlin criteria, is essential for enhancing the comparability and reliability of ARDS research and for advancing clinical practice.

Furthermore, it was noted that some articles did not differentiate ARDS severity, merely reporting its presence or absence without subdividing patients according to severity. This lack of specificity in severity classification may influence the interpretation of data and the application of ventilatory strategies in clinical practice. The variability in classifications and intervention times highlights the need for a standardised consensus to assess the severity of ARDS and to guide the timing of ventilatory interventions, thus ensuring better adaptation to the clinical needs of each patient and facilitating comparison of results between studies.

Through the analysis of reported outcomes, a distinct cleavage can be observed between those related to the intensive care unit (ICU) time period and the remainder of the recovery process. Over 40% of the reviewed articles focus on ICU length of stay and ICU mortality rates, highlighting a predominant emphasis on the early stages of recovery. This focus results in a paucity of evidence regarding outcomes in the more advanced stages post-ICU discharge and overall hospital release. Latronico et al. reported an absolute increase of 25% in late mortality for ARDS patients admitted to the hospital compared to non-hospitalized patients [35]. Understanding the entire recovery process is paramount for delivering accurate and comprehensive treatment. The Clinical Frailty Scale (CFS), developed in 2005, assesses the overall frailty of patients before ICU admission [36]. The CFS is currently used to predict outcomes such as 30-day mortality in ICU patients [37]. However, there is a critical need to better understand and plan for outcomes related to the later stages of recovery in critically ill ARDS patients. Developing tools that address long-term recovery outcomes, similar to the CFS’s role in acute stages, is essential. Addressing this gap will ensure a comprehensive approach to patient care, extending beyond the ICU to include long-term recovery.

Temporality related to mortality is reported in 35% of the articles, and the duration of ventilation is discussed in 43% of them. Understanding temporality, particularly concerning mortality, is crucial for identifying the underlying causes. A comprehensive time-line is needed from before the treatment application to its varied consequences. However, the focus in the literature is predominantly on the acute stage of the syndrome. Given that temporality extends beyond the hospital setting, this narrow perspective may hinder critical treatment decisions. Addressing lifelong impairment is essential when providing care to ARDS patients, ensuring that treatment strategies account for the extended timeline of the patient’s condition. Studies by Herridge et al. have highlighted the long-term sequelae of ARDS, emphasising the necessity of considering extended recovery periods [38]. Furthermore, future research should consider detailing the specific timing of each ventilatory therapy to evaluate the contribution of each in managing ARDS. Understanding temporality is also pivotal for decision-making regarding the management of ARDS and for generating strategies aimed at optimizing resource allocation and reducing ICU length of stay. Prospective studies are needed to enable causal analyses of the timing of therapeutic applications and their clinical outcomes.

Another vital parameter in assessing a patient’s overall condition is their quality of life. Following ICU and hospital stays, quality of life becomes a significant concern for ARDS patients. Notably, none of the articles in this scoping review reported data on quality-of-life outcomes. The Post-Intensive Care Unit Syndrome (PICS) underscores the importance of considering quality of life when developing treatment strategies, even for younger patients, as evidenced by Davidson et al. [39]. The study by Szymczak et al. highlights that pre-ICU factors, including obesity, physical and mental comorbidities, and smoking status, have significant implications for the long-term recovery process and mental health-related quality of life (HRQoL) of ARDS survivors [40]. Their findings show that pre-existing mental health problems, such as anxiety, depression, and PTSD, are particularly impactful, suggesting that early identification and intervention could improve long-term mental HRQoL outcomes for ARDS patients. While the current focus is primarily on the patient’s condition before and during hospitalisation, there is an urgent need to enhance our understanding of post-hospitalization conditions. This knowledge is crucial for informing appropriate and effective care strategies that extend beyond the hospital environment.

The results of this review mainly point to articles published from 2015 onwards. This probably coincides with a greater interest in and more tools for the treatment of ARDS after the guidelines associated with the Berlin consensus of 2012 [5]. In addition, the need to address this type of pathology during the COVID-19 pandemic may also have influenced the increase in this type of publication in recent years, as this became one of the important causes of hypoxemic respiratory failure and ARDS [4]. Likewise, articles that involve patients diagnosed with COVID-19 were included in this study, so it must be considered that some additional considerations to the classically used management of respiratory failure are included in the most current literature.

The quality-of-life outcome has been underreported in the reviewed articles, since, in this case, the reporting of physiological outcomes such as oxygenation through PaO_2_/FiO_2_, and lung mechanics, among others, was probably considered more relevant. This may be because the main focus in these articles was linked to the acute phase of ARDS management. However, the focus of outcomes reported in the ICU has changed in recent years; Classically, greater importance was given to physiological results with respect to functional ones, but these do not necessarily translate into a clinical effect or in the patient’s function, for this reason it is sought that the interventions and measurements are relevant to patients, their families and society, which has resulted in the development of so-called “patient-centred outcomes” [41].

Several studies historically mention that the ventilatory management of ARDS has to do with the use of low tidal volumes, and trends towards higher PEEP levels, such as the randomised controlled trial of the ARDS Network from 2000. In turn, specialists such as Luciano Gattinoni, who analysed studies such as “LOV”, “EXPRESS” and “AL-VEOLI”, in which, despite obtaining inconclusive results, the use of high levels of PEEP compatible with plateau pressures of 28–30 cm-H_2_O is recommended, in addition to a tidal volume of 6 ml/kg of predicted weight [42]. Although in the articles included in this review, the foregoing is mentioned, no categorical recommendations are made, which may be due to the heterogeneity and individuality of the patients, since, although there are standard recommendations that are applied in a basal way, constant evaluation is still the greatest guideline to intervene.

Manoeuvres such as prone, the use of NMB, and alveolar recruitment manoeuvres are mentioned in the various articles included in this review, which is consistent with both ventilatory and non-ventilatory recommendations at the international level regarding the management of ARDS, especially in moderate and/or severe stages [2].

## Conclusion

This scoping review identified a variety of commonly employed techniques, including HFNC, NIV, and IMV. Each technique has specific characteristics that influence its application in different ARDS contexts, such as invasiveness, ability to provide pressure support, and potential impact on patient comfort and long-term outcomes.

The review also highlighted the critical role of temporality in ventilatory support for ARDS. While the included studies documented the timing of interventions, temporality was not often the primary focus of investigation. This suggests that further research is needed to establish clear guidelines on the optimal timing and sequencing of ventilatory techniques, considering factors such as disease severity, patient response, and the potential for long-term complications like PICS.

In addition to the techniques themselves and their temporality, the review revealed a need for greater emphasis on patient-centred outcomes, including quality of life and functional status, in ARDS management and research.

## Appendix Supplementary material

### Key words research

**Table:**

**Data bases**	**Key words combination**	**Articles retrieved**
Pubmed 20/10/2023 15h47 UTC+2	(((“Adult”[Mesh] OR “Adult” OR “Patients”[Mesh] OR “Patients”) AND (“Intensive Care Units”[Mesh] OR “Intensive Care Units”) AND (“Respiratory Distress Syndrome”[Mesh] OR “Respiratory Distress Syndrome” OR “Acute Respiratory Distress Syndrome” OR “ARDS”)) AND ((“Respiratory Therapy”[Mesh] OR “Respiratory Therapy” OR “Respiration, Artificial”[Mesh] OR “Respiration, Artificial”) OR (“Therapeutics”[Mesh] OR “Therapeutics”) OR “Treatment” OR (“Noninvasive Ventilation”[Majr] OR “Noninvasive Ventilation” OR “Intubation, Intratracheal”[Majr] OR “Intubation, Intratracheal” OR “Intubation”[Mesh] OR “Endotracheal Intubation” OR “High Flow Nasal Canula” OR “HFNC” OR “High Flow Oxygen Therapy” OR “HFOT”)) AND (“Time”[Mesh] OR “Time” OR “Timing” OR “Time-to-Treatment”[Mesh] OR “Time-to-Treatment” OR “Time Management”[Mesh] OR “Time Management”)) [FILTERS: 10 YEARS - HUMAN - ADULTS +19]	297

Science direct 25/10/2023 18h49 UTC+2	“intensive care unit” AND (“Acute Respiratory Distress Syndrome” OR “ARDS”) AND (“Ventilation” OR Noninvasive Ventilation OR Intubation OR “High Flow Nasal Cannula” OR High Flow Oxygen Therapy) AND time	61
	*Year(s)*: 2013–2023; research articles	

EBSCO 26/10/2023 16h51 UTC+2	(((“Adult”[Mesh] OR “Adult” OR “Patients”[Mesh] OR “Patients”) AND (“Intensive Care Units”[Mesh] OR “Intensive Care Units”) AND (“Respiratory Distress Syndrome”[Mesh] OR “Respiratory Distress Syndrome” OR “Acute Respiratory Distress Syndrome” OR “ARDS”)) AND ((“Respiratory Therapy”[Mesh] OR “Respiratory Therapy” OR “Respiration, Artificial”[Mesh] OR “Respiration, Artificial”) OR (“Therapeutics”[Mesh] OR “Therapeutics”) OR “Treatment” OR (“Noninvasive Ventilation”[Majr] OR “Noninvasive Ventilation” OR “Intubation, Intratracheal”[Majr] OR “Intubation, Intratracheal” OR “Intubation”[Mesh] OR “Endotracheal Intubation” OR “High Flow Nasal Canula” OR “HFNC” OR “High Flow Oxygen Therapy”	79
Academic search CINHAL Sportsdiscuss EJournals		
